# The role of total quality management in enhancing customer satisfaction in Gulf Cooperation Council (GCC) countries

**DOI:** 10.1016/j.mex.2024.102854

**Published:** 2024-07-06

**Authors:** Saud Alsaqer, Ihab Katar, Abdelhakim Abdelhadi

**Affiliations:** Master in Engineering Management, College of Engineering, Prince Sultan University, Riyadh 11586, Saudi Arabia

**Keywords:** Total quality management, Customer satisfaction, Employee engagement, Process management, Customer focus, Continuous improvement practices, Total Quality Management

## Abstract

This study examined the role of Total Quality Management (TQM) practices, specifically continuous improvement, customer focus, process management, and employee engagement, in advancing sustainability and enhancing customer satisfaction in the telecommunications sector, focusing on three firms in Gulf Cooperation Council (GCC) countries. Secondary quantitative data from quarterly reports (2019–2023) were analyzed using descriptive, correlation, and regression methods with STATA software.

• The findings indicated an increase in net promoter score over the study period, reflecting firms' commitment to addressing changing customer needs.
• Employee engagement and process management had a positive and statistically significant effect on customer satisfaction.
• Integrating TQM practices to enhance customer satisfaction in telecommunications.

## Graphical abstract

**Figure:**
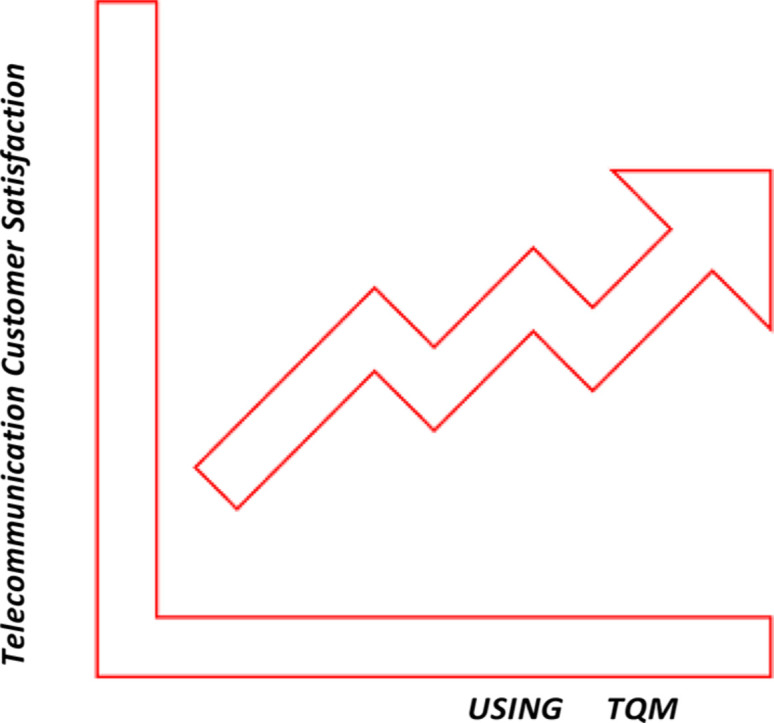

Specifications table

**Table:** 

Subject area:	Engineering
More specific subject area:	Customer Satisfaction
Name of your method:	Total Quality Management
Name and reference of original method:	NA.
Resource availability:	NA.

## Background

In the current business environment, organizations face intense competition from local and global firms that offer outstanding products and services in terms of affordability and quality to customers [1]. As a result, businesses are faced with the need to meet and satisfy customer expectations by aligning their business operations with customer satisfaction. To realize customer satisfaction, businesses are attempting to implement total quality management (TQM), which is an overarching organizational strategy for continuously improving products, services, and processes to realize business efficiency and satisfy consumer needs [2]. By adopting total quality management, firms are able to improve their operations, enhance the quality of products and services, and focus on their customers. According to Zaid et al. [3], total quality management demands that organizations adjust their quality to the level demanded by customers so as to remain competitive. A growing number of companies have embraced TQM as a key strategy for improving customer retention, customer satisfaction, and firm performance. Firms that have won quality awards generally outperform other companies with regard to customer satisfaction, income measures, and market value [2].

In the Kingdom of Saudi Arabia, the telecommunication industry is highly competitive due to the large number of mobile service providers that also face difficulties in satisfying the diverse needs of customers. For instance, in Q3 of 2020, the total number of mobile service complaints amounted to 14,720 [4]. Zain accounted for the highest number of mobile voice complaints, with 30 complaints per 100,000 subscribers, followed by Saudi Telecom Company (STC), Mobily, Lebara Mobile, and Virgin Mobile. The complaints were about financial obligations, poor services, and billing. Due to these challenges, there is a need to deliver high-quality services so as to keep current customers, attract new ones, and gain competitive advantages. Some of these companies have adopted TQM practices such as continuous improvement, customer focus, risk management, and employee engagement to improve the quality of their products and services.

From existing literature, few studies have been performed on the influence of TQM on customer satisfaction in the GCC countries, with available studies focusing mainly on other sectors and countries. For example, Zaid et al. [3] focused on the Palestine health sector, Noori [2] examined the Indian retail sector, while Nunkoo et al. [5] focused on the UK hotel industry. Nguyen and Nagase [6] focused on Vietnam's healthcare facilities. The findings from these studies may not be relevant in the context of telecommunication firms in the GCC. Additionally, existing studies have found inconsistent results on the relationship between TQM dimensions and customer satisfaction [7]. As such, this study seeks to fulfill these research gaps by exploring the influence of TQM practices such as process management, customer focus, process management, continuous improvement, and employee engagement on customer satisfaction in the context of telecommunication firms operating in the GCC Total Quality Management (TQM) initiatives significantly influence customer satisfaction through various dimensions, including process management, customer focus, continuous improvement, and employee engagement. Process management ensures efficient business operations by analyzing and improving current systems. Studies have shown a positive relationship between process management and customer satisfaction. For example, Ooi et al. [8] found that effective process management in Malaysian SMEs enhanced customer satisfaction by streamlining operations and improving product delivery. This systematic approach identifies bottlenecks and areas for improvement, ultimately boosting organizational competitiveness and customer satisfaction. Additionally, Al-Diabat [9] emphasized the mediating role of Business Process Management (BPM) in enhancing customer loyalty through CRM initiatives in the Jordanian telecommunications sector. This study demonstrated that aligning business processes with customer needs significantly improves customer loyalty, reinforcing the importance of BPM in maintaining competitive advantage and achieving high customer satisfaction.

Customer focus involves centralizing customer needs in management decisions and business development. Noori [2] found that customer-centric approaches significantly improve customer satisfaction by addressing needs and preferences effectively. Firms that understand and respond to customer requirements tend to have higher satisfaction rates. This involves maintaining close relationships with customers, gathering information about their needs, and demonstrating responsiveness. Fader [10] further emphasizes that customer centricity is not merely about being customer-friendly but strategically aligning company offerings with the desires of the most valuable customers. This approach ensures long-term profitability and competitive advantage, as illustrated by companies like Nordstrom, which have built reputations for exceptional customer service by continuously adapting to customer feedback and preferences. Continuous improvement, described as ongoing enhancements in product and service quality, ensures that customer expectations are met, resulting in increased satisfaction and loyalty. Continuous improvement practices involve both incremental and breakthrough improvements, ensuring that new and improved products and services are consistently offered to customers [11]. Hassan et al. [12] highlighted that continuous improvement initiatives boost organizational performance and customer satisfaction by constantly refining processes to better meet customer needs. Furthermore, a study by [13] outlines how continuous improvement processes can significantly enhance an organization's compliance with rules and regulations. It emphasizes that continuous improvement in compliance programs involves regular internal audits, control testing, and evolving updates to policies and procedures. This proactive approach helps organizations stay ahead of compliance issues by identifying and addressing potential nonconformities before they escalate employee engagement and empowerment are critical for the success of TQM initiatives. Engaged employees, who are actively involved in decision-making and feel committed to their work, contribute significantly to customer satisfaction. Kim [11] demonstrated that employee empowerment through training and resource provision leads to a positive work environment and better customer satisfaction. When employees are empowered to make decisions and address quality issues, they are more likely to deliver high-quality service, resulting in higher customer satisfaction. Overall, effective TQM practices across these dimensions are essential for improving customer satisfaction. In addition, Sweis et al. [14] employee empowerment had a positive effect on Saudi Arabian healthcare workers. Notably, capacity-based projects, training opportunities, coordination, and collaboration improved employees' levels of involvement and empowerment, resulting in increased satisfaction with their jobs and, subsequently, client satisfaction. Supporting the findings, Chen et al. [15]. showed that employee empowerment by Taiwanese security firms positively and significantly impacted client satisfaction. This was due to training and the provision of adequate resources that cultivated a positive work environment to address the needs of clients. Consistently, Abdull et al. [16] found that staff engagement and constant development have a positive effect on the levels of employee and customer satisfaction in the Qatar Ministry of Interior. Findings from their study helped to suggest effective strategic decisions that firms can use to improve employee efficiency and customer satisfaction. However, a study by Ooi et al. [8] showed that human resource focus, which entails employee involvement and empowerment, has an insignificant impact on customer satisfaction. This could be attributed to a lack of clarity in empowerment guidelines, which makes employees struggle when making decisions that align with customer expectations.

## Method details

This study deployed a quantitative research design to explore the impact of TQM practices on customer satisfaction in the GCC for three telecommunication firms. Quantitative design is considered appropriate as it takes an objective approach to gain insights from collected data through statistical analysis. The data collection was carried out by contacting multiple telecommunications firms in the GCC obtaining quarterly reports from 2019 to 2023. Firms were strategically sampled based on market presence, operational scale, geographic diversity, and data availability. This approach ensured a comprehensive dataset reflecting industry-wide trends in TQM practices. By engaging these firms directly, we gathered a robust foundation for analyzing the impact of TQM on customer satisfaction, allowing us to draw meaningful insights relevant to our research objectives.

**Data collection and preparation process:**

• Data Access and Extraction: multiple telecommunications firms were contacted in the GCC and requested their performance reports. While some firms responded positively and provided the necessary reports, others did not reply, which influenced our final sample. For the firms that provided the reports, relevant data was manually extracted. Key performance indicators such as Net Promoter Score (NPS), training hours per employee, average internet download speed, first call resolution percentage, and the number of escalated complaints per 100,000 subscribers were identified and recorded.
• Data Verification: To ensure accuracy, the extracted data was cross-verified with other available sources and reports. This step was crucial for maintaining consistency across different firms' reporting standards.
• Data Standardization: The data was then standardized to align with uniform metrics and formats. This involved converting different units of measurement to a common standard where necessary and normalizing the data to ensure comparability.

**Fig. 1:**
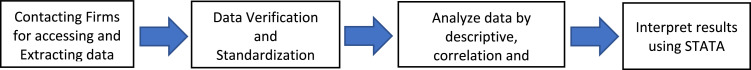
Data collection and preparation process.

Fig. 1 briefs the data collection and preparation process.

Specific indicators were selected to link both dependent and independent variables effectively. The Net Promoter Score (NPS) was selected as the dependent variable representing customer satisfaction due to its robust nature in measuring customer loyalty and satisfaction, providing a clear metric for assessing customer perceptions of a company's services [17]. Employee engagement was linked to training hours per employee, as training is a crucial component of employee development and engagement. The number of training hours reflects the company's investment in its workforce, enhancing employees' skills, motivation, and overall engagement. Engaged employees are more likely to deliver high-quality service, directly impacting customer satisfaction [18]. Process management was associated with the average internet download speed. In the telecom sector, efficient process management ensures optimal network performance, directly influencing download speeds. Higher download speeds are a tangible outcome of well-managed processes, leading to enhanced customer experiences and satisfaction [19]. Customer focus was linked to the first call resolution (FCR) percentage. FCR is a critical measure of a company's ability to resolve customer issues during the first interaction, reflecting responsiveness and efficiency. A higher FCR indicates effective customer-focused practices, leading to increased customer satisfaction [20]. Continuous improvement was measured by the number of escalated complaints per 100,000 subscribers. This metric highlights the company's efforts to reduce service issues and continuously enhance quality. Fewer escalated complaints indicate effective continuous improvement practices, leading to higher customer satisfaction [21].

## Method validation

This study deployed a quantitative research design to explore the impact of TQM practices on customer satisfaction in the GCC for three telecommunication firms. Quantitative design is considered appropriate as it takes an objective approach to gain insights from collected data through statistical analysis. The secondary data was collected from the quarterly report of the three firms sampled in the study for the period from 2019 to 2023. Specific indicators were selected to link both dependent and independent variables effectively. The Net Promoter Score (NPS) was selected as the dependent variable representing customer satisfaction due to its robust nature in measuring customer loyalty and satisfaction, providing a clear metric for assessing customer perceptions of a company's services [17]. Employee engagement was linked to training hours per employee, as training is a crucial component of employee development and engagement. The number of training hours reflects the company's investment in its workforce, enhancing employees' skills, motivation, and overall engagement. Engaged employees are more likely to deliver high-quality service, directly impacting customer satisfaction [18]. Process management was associated with the average internet download speed. In the telecom sector, efficient process management ensures optimal network performance, directly influencing download speeds. Higher download speeds are a tangible outcome of well-managed processes, leading to enhanced customer experiences and satisfaction [19]. Customer focus was linked to the first call resolution (FCR) percentage. FCR is a critical measure of a company's ability to resolve customer issues during the first interaction, reflecting responsiveness and efficiency. A higher FCR indicates effective customer-focused practices, leading to increased customer satisfaction [20]. Continuous improvement was measured by the number of escalated complaints per 100,000 subscribers. This metric highlights the company's efforts to reduce service issues and continuously enhance quality. Fewer escalated complaints indicate effective continuous improvement practices, leading to higher customer satisfaction [21].

Table 1 shows the specific data collected for each of the variables in the study. The data was statistically analyzed using descriptive statistics and inferential statistics. Descriptive statistics was used to summarize the data. Conversely, inferential statistics, specifically correlation and regression analysis, was used to assess the correlation and relationship between the study variables. Correlation analysis is crucial when determining the strength and direction of the relationship between two variables (TQM and Customer satisfaction) [22]. Regression analysis was to indicate the causal relationships between research variables and this included evaluating the statistical significance of the relationship [22]. Data was analyzed using STATA software given its ability to analyze panel data. The multiple regression analysis was run with Customer satisfaction (Net promoter score) as the dependent variable and customer focus, process management, employees' engagement, and continuous improvement practices as dependent variables. The regression model is summarized below;

NPSit=β0+β1EEit+β2.PMit+β3CIP+β4CF+εit

**Table 1:** Research variables.

Nature of Variable	Research variable	Symbol	Measure of variable
Dependent Variable	Customer Satisfaction	NPS	Net promoter score (NPS.)
Independent Variable	Employee engagement	EE.	Training hours per employee
Independent Variable	Process management	PM	Average internet download speed (Mb/s)
Independent Variable	Customer Focus	CF	First call Resolution (%)
Independent Variable	Continuous improvement practices	C.I.P.	The number of escalated complaints per 100k subscribers

The symbols are as summarized below:

• β0 is the regression Constant, β1 to β4 are the regression coefficients
• εit - is the error term
• *i*= represents the three (3) telecom firms from the 1st to the 3rd, while t is the time in quarters, from the first quarter in 2019 to the last quarter in 2023 (1st to 20th).

## Results

This section critically discussed the research findings and results on the impact of TQM on customer satisfaction in the GCC telecommunication sector using the three sampled firms. The findings were presented in accordance with the research objectives, as shown in Table 2.

**Table 2:** Descriptive statistics.

Variable	Obs	Mean	Std. Dev.	Min	Max
N.P.S.	60	77.550	4.176	69.000	85.000
Employee Engagement	60	32.684	7.953	15.500	50.450
Continuous improvement practices	60	13.633	2.834	8.000	21.000
Customer focus	60	77.902	3.137	70.840	81.972
Process management	60	102.860	7.729	89.300	121.000

From Table 3, the NPS variable has an average of 77.55, an indication of high customer satisfaction with the products and services provided by the telecommunication firms. The standard deviation (4.176) shows a significant difference in the NPS around the mean.

**Table 3:** Correlation matrix.

	N.P.S.	Employee engagement	Continuous Improvement	Customer focus	Process management
NPS	1				
Employee engagement	0.9306	1			
Continuous Improvement	−0.2748	−0.2095	1		
Customer focus	0.5779	0.5458	−0.3818	1	
Process management	0.8757	0.8541	−0.2629	0.4239	1

As for employee engagement, the average level of training hours per employee for firms in the telecommunication sector was 32.684. With a standard deviation of 7.953, there is a high level of variation in employee engagement in the sector. Focusing on the continuous improvement practices variable, the findings indicated an average of 13.633 customer complaints per 100,000 subscribers. A standard deviation of 2.834 indicated a high level of difference between the continuous improvement practices of firms in the telecom sector.

The customer focus variable posted an average of 77.902 % first call resolution, with a standard deviation of 3.137. The standard deviation indicates that there is a statistically significant difference between the customer focus by the firms in the telecom sector.

Finally, process management for the three telecommunication firms had an average internet download speed of 102.860 Mb/s with a standard deviation of 7.729. The high standard deviation indicates a significant difference in the process management of the firms in the telecom sector.

**Fig. 2:**
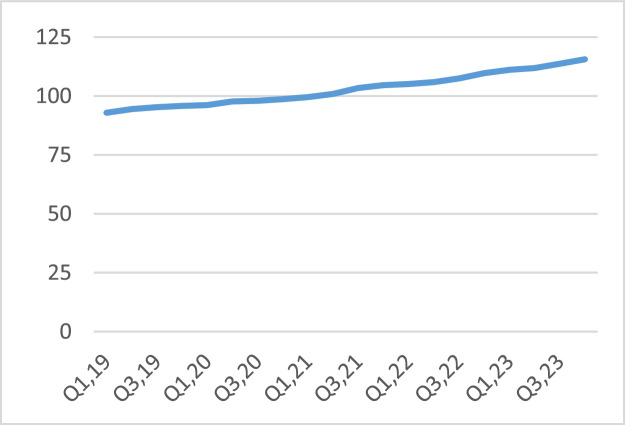
Average internet download speed.

Focusing on trends in the various variables in the study, the findings indicated that the average internet download speed for the sector had consistently risen from 92.87 MB/S in the first quarter of 2019 to 115.57 MB/S in the last quarter of 2023, as shown in Fig. 2. Being a proxy of process management, the findings basically pointed to enhanced processes by the firms in the sector, which has enhanced the download speed. This is ultimately bound to enhance customer satisfaction as they find the internet speed very friendly and consistently increasing. This locks the customers to the brand.

**Fig. 3:**
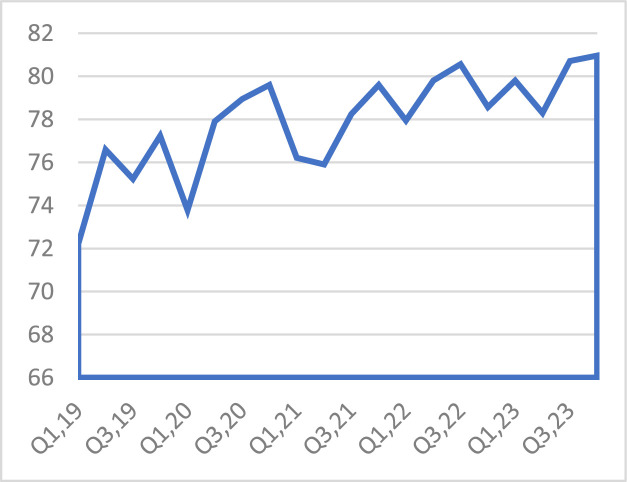
Average first call resolution (%).

With regards to customer focus, which was proxied by first call resolution (%) in the GCC telecom sector, this sharply fluctuated in the study period. Fig. 3 shows the trend in first call resolution (%). Overall, this has risen from 72.21 % to 80.96 % which depicts an enhanced customer focus over the period.

**Fig. 4:**
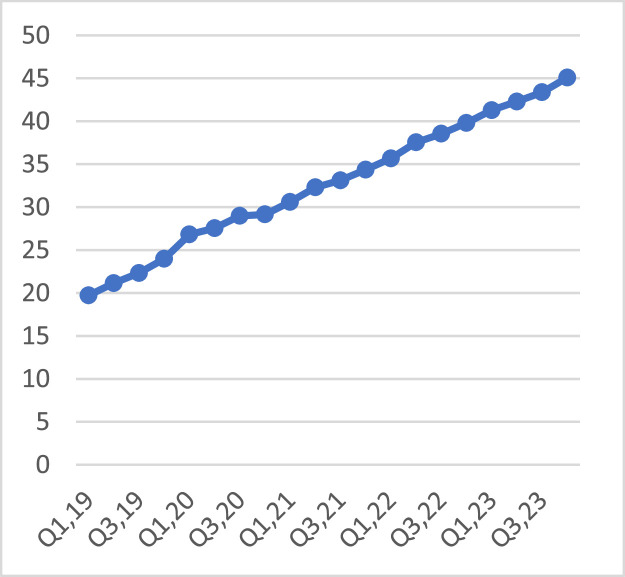
Training hours per employee.

**Fig. 5:**
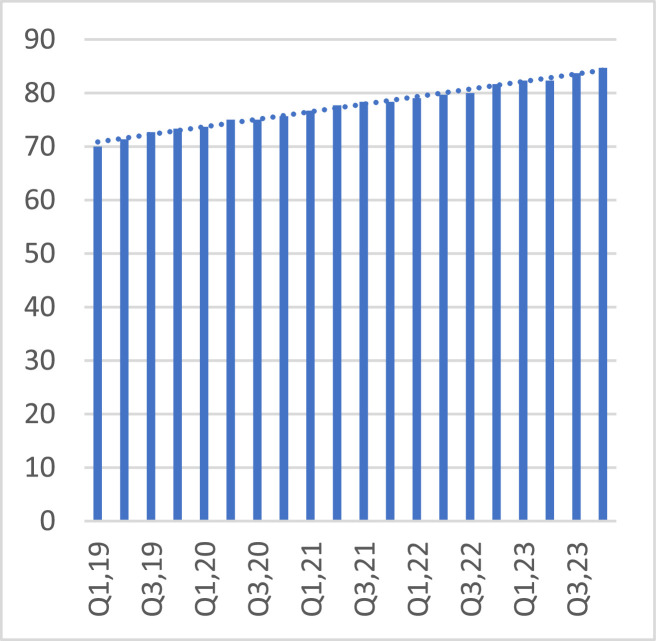
Net promoter score.

Delving into the trend on employee engagement (training hours per employee, Fig. 4) and net promoter score (customer satisfaction), Fig. 5, the averages for the sector for each of the two variables rose. An increment in employee engagement basically infers the empowerment of employees with skills pertinent to steering productivity and innovation. This ultimately is bound to enhance customer satisfaction.

The study findings further indicated a fluctuation in the number of escalated complaints per 100,000 subscribers in the telecom sector. However, overall, the number of escalated complaints dipped from 15.67 in the first quarter of 2019 to 11.33 in the last quarter of 2023. This means the complaints declined, which is a consequence of a continuous improvement in the telecom sector's practices. This includes robust policies and practices put in place to effectively address gaps in the services that have seen an escalation of complaints in the past.

With regard to the correlation analysis in Table 3, there is a strong positive correlation (0.9306) between employee engagement and customer satisfaction (NPS). Process management was also found to have a statistically significant positive correlation with net promoter score (Customer satisfaction). The correlation coefficient was found to be 0.8757. In between the independent variables, process management was found to have a statistically significant positive correlation with employee engagement (correlation coefficient of 0.8541). This could be explained by the intense involvement of employees during the enhancement of processes in the telecom sector.

The findings in Table 4. indicated an R squared of 0.9006. This is to mean that the independent variables explain 90.06 % of the variation in the dependent variable (customer satisfaction). This basically infers the indispensable value of total quality management and, precisely the dimensions of TQM considered in the study in shaping customer satisfaction in the telecom sector. Moving forward F statistic was found to have a p-value of 0.000. This means that the model is fit. Further, it means that the TQM dimensions considered in the study were found to have a statistically significant effect on customer satisfaction.

**Table 4:** Regression analysis.

Source	SS.	df	Ms		Number of obs	60
					F(4, 55)	124.56
Model	926.5647	4	231.6412		Prob > *F*	0.000
Residual	102.2853	55	1.8597		R-squared	0.9006
					Adj R-squared	0.8934
Total	1028.85	59	17.4381		Root MSE	1.3637
NPS	Coef.	Std. Err. t	t	*P* > *t*	[95 % Conf.	Interval]
Training hours	0.3168	0.0473	6.7	0.0000	0.2220	0.4117
Number of escalated complains	−0.0386	0.0693	−0.56	0.5800	−0.1775	0.1003
First call resolution	0.1440	0.0724	1.99	0.0520	−0.0011	0.2891
Average Internet Speed	0.1662	0.0454	3.66	0.0010	0.0752	0.2571
_cons	39.4101	7.1594	5.5	0.0000	25.0623	53.7579

### Effect of employees engagement on customer satisfaction

With a positive coefficient at 0.3168 and a P value of 0.000 at a 5 % significance level, the findings indicate that employee engagement has a statistically significant positive effect on customer satisfaction. The P value is lower than the 5 % significance level. Enhanced employee engagement is pertinent in that it fosters productivity and innovation emanating from skills development. Through training, the employees are able to gain in-depth knowledge about telecommunication products and services. They thus are able to work on modalities of enhancing systems, processes, and procedures aimed to enhance service delivery to the customers. Besides, training the employees has a positive effect on their attitude and confidence. These findings corroborated with those of Sweis et al. [14], which found that employee engagement through capacity-based projects and training opportunities significantly increases employee satisfaction/productivity and this ultimately translates into enhanced customer satisfaction.

### Effect of continuous improvement practices on customer satisfaction

A negative coefficient of 0.0386 and a P value of 0.58 at a 0.05 significance level show that continuous improvement practices have no statistically significant effect on customer satisfaction in the GCC telecommunication sector. The P value is higher than the 5 % significance level. This means that increasing continuous improvement practices by telecommunication firms does not significantly influence consumer satisfaction. This is despite the value of continuous evaluation of practices to identify gaps and to execute strategies that seal the gaps to enhance customer satisfaction. The study finding is consistent with [23], which found no significant association between continuous improvement and customer satisfaction for students at the University of Sultan Zainal Abidin, Malaysia. This was attributed to misalignment with student needs and lack of students' involvement when improving library books and services.

### Effect of customer focus on customer satisfaction

A positive coefficient of 0.1440 and a P value of 0.0520 at a 5 % significance level indicates that customer focus has no statistical effect on customer satisfaction in the GCC telecommunication sector. The P value is higher than the 5 % significance level (0.05). This means that an increase in customer-focus practices by telecommunication firms will not necessarily result in a statistically significant rise in customer satisfaction. However, it cannot be discounted that when customer service calls are resolved at initial contact without calling for further follow-ups, it leads to immense customer satisfaction. This builds trust and loyalty to the brand. When customer service calls are not resolved on time, the customers may shift to other brands that they deem responsive.

### Effect of process management on customer satisfaction

A positive coefficient of 0.1662 and a p-value of 0.001 (lower than 0.05 significance level) indicates that process management has a statistically significant and positive effect on customer satisfaction in the GCC telecommunication sector. This shows that improvements in process management by telecommunication firms increase customer satisfaction. In essence, an increase in the average internet download speed of up to 100 Mb/s would mean that customers enjoy faster access to online content, quick software updates, and improved online experience when accessing webpages, videos, or images. Additionally, an increase in download speeds would improve the streaming quality of video content without buffering, thus creating a more enjoyable viewing experience and satisfaction with telecommunication services. Accordingly, improvement in the processes by the telecommunication firms would improve customers' experience as well as their satisfaction. In line with these findings, previous research have shown that improvement in process management has a significant and positive influence on customer satisfaction [8,17]. Accordingly, well-managed processes that are designed to meet or exceed customer expectations result in improved service quality, which positively influences customer satisfaction.

## Limitations

The analysis revealed several key insights as the findings underscore the pivotal role of employee engagement in enhancing customer satisfaction. In the fiercely competitive telecom sector, firms must invest in continuous employee training to foster creativity and productivity amidst rapid technological advancements. Such investments lead to improved service delivery through streamlined processes, effectively addressing customer complaints and service gaps, thereby positively influencing customer satisfaction.

Process management, measured by average internet download speed, also emerged as a critical factor with a statistically significant positive effect on customer satisfaction. Optimizing business processes enhances operational efficiency, leading to stable networks and faster download speeds, which directly contribute to customer satisfaction. Therefore, it is essential for telecom firms to continuously educate their employees on the importance of efficient processes in meeting customer expectations. Interestingly, the study found that continuous improvement practices did not have a statistically significant effect on customer satisfaction. Despite this, the value of continuous improvement in the telecom sector should not be underestimated. Constant efforts to enhance services, processes, and products are crucial in retaining customers amidst intense competition. Telecom firms must identify areas needing improvement and allocate resources to address them, as competitors could exploit these weaknesses.

Furthermore, the study revealed that customer focus alone does not have a statistically significant impact on customer satisfaction. While customer centricity is vital, other factors, such as organizational culture, leadership, and employee engagement, play more significant roles. However, in a sector characterized by intense competition and sensitive customers, understanding and addressing customer needs remain crucial. This necessitates substantial investments in research and development to tailor services to customer preferences, ensuring that firms do not lose customers to competitors due to unmet needs. However, several limitations and challenges must be considered for a comprehensive understanding of the research. The sample size, although representative, remains limited due to the difficulty in accessing comprehensive data from a broader range of firms. This limitation might affect the generalizability of our findings across the entire telecommunications industry in the GCC region. The strategic criteria for selecting participating firms may introduce selection bias, as firms with well-established TQM practices and better-documented customer satisfaction metrics were more likely to be included in our study. This could mean that the findings may not fully represent firms with less mature TQM practices. Maintaining confidentiality for participating firms was necessary but limited the transparency of the study. The inability to disclose specific firm names and details might reduce the study's replicability by other researchers aiming to validate or extend our findings. Our study relied on secondary data from quarterly reports provided by the firms. Variations in reporting standards, data collection methods, and report comprehensiveness across different firms could introduce inconsistencies in the dataset, potentially affecting the accuracy of our analysis and the robustness of our conclusions. The data spans from 2019 to 2023, a period marked by significant global events such as the COVID-19 pandemic. These events might have uniquely influenced customer satisfaction and TQM practices in ways atypical of normal operational conditions. These external factors need consideration when interpreting our results. The study focuses exclusively on the telecommunications industry within the GCC countries. While providing valuable insights specific to this context, the findings may not directly apply to telecommunications industries in other regions with different market dynamics, regulatory environments, and customer expectations. Other variables influencing customer satisfaction and TQM outcomes may not have been captured in the available data. Factors such as market competition, regulatory changes, and technological advancements might also significantly shape the relationship between TQM initiatives and customer satisfaction. Acknowledging these limitations, we aim to provide a balanced perspective on our findings. Future research should address these limitations by expanding the sample size, including a broader range of firms, and exploring additional variables that might influence the outcomes. Despite these limitations, our study offers valuable contributions to understanding the effects of TQM initiatives on customer satisfaction in the telecommunications industry within the GCC region.

To enhance customer satisfaction, the study recommends several strategic actions for telecom firms. First, investments in advanced Customer Relationship Management (CRM) systems are essential. Utilizing data analytics and AI can help firms understand customer behaviors and preferences, allowing for personalized services and proactive issue resolution. Second, improving employee engagement through regular training programs is critical. Engaged employees, well-versed in the latest technological advancements, are more likely to provide exceptional customer service and handle complex inquiries effectively. Finally, establishing effective feedback loops is crucial. Capturing real-time customer feedback and integrating it into continuous improvement processes ensures that services are consistently refined based on customer input, fostering an environment of ongoing enhancement and customer satisfaction.

## Author statement

All authors have read and agreed to the published version of the manuscript.

## Ethics statements

NA.

## CRediT authorship contribution statement

**Saud Alsaqer:** Conceptualization, Methodology, Software, Validation, Formal analysis, Investigation, Resources, Data curation, Writing – original draft, Writing – review & editing. **Ihab Katar:** Conceptualization, Software, Validation, Formal analysis, Investigation, Resources, Data curation, Writing – original draft, Writing – review & editing, Supervision. **Abdelhakim Abdelhadi:** Conceptualization, Software, Validation, Formal analysis, Investigation, Resources, Data curation, Writing – original draft, Writing – review & editing, Supervision.

## Declaration of competing interest

The authors declare that they have no known competing financial interests or personal relationships that could have appeared to influence the work reported in this paper.

## Data Availability

Data will be made available on request.
